# Dl-3-n-Butylphthalide Alleviates Hippocampal Neuron Damage in Chronic Cerebral Hypoperfusion *via* Regulation of the CNTF/CNTFRα/JAK2/STAT3 Signaling Pathways

**DOI:** 10.3389/fnagi.2020.587403

**Published:** 2021-01-13

**Authors:** Wenxian Li, Di Wei, Zheng Zhu, Xiaomei Xie, Shuqin Zhan, Ru Zhang, Guilian Zhang, Li’an Huang

**Affiliations:** ^1^Department of Neurology, The Second Affiliated Hospital, Xi’an Jiaotong University, Xi’an, China; ^2^Department of Urology, Xijing Hospital, The Fourth Military Medical University, Xi’an, China; ^3^Department of Internal Medicine, Division of Hematology/Oncology, University of California Davis, Sacramento, CA, United States; ^4^Department of Neurology, The First Affiliated Hospital, Jinan University, Guangzhou, China

**Keywords:** chronic cerebral hypoperfusion, neuronal death, vascular cognitive impairment, CNTF signaling, Dl-3-n-butylphthalide

## Abstract

Chronic cerebral hypoperfusion (CCH) contributes to cognitive impairments, and hippocampal neuronal death is one of the key factors involved in this process. Dl-3-n-butylphthalide (D3NB) is a synthetic compound originally isolated from the seeds of *Apium graveolens*, which exhibits neuroprotective effects against some neurological diseases. However, the protective mechanisms of D3NB in a CCH model mimicking vascular cognitive impairment remains to be explored. We induced CCH in rats by a bilateral common carotid artery occlusion (BCCAO) operation. Animals were randomly divided into a sham-operated group, CCH 4-week group, CCH 8-week group, and the corresponding D3NB-treatment groups. Cultured primary hippocampal neurons were exposed to oxygen-glucose deprivation/reperfusion (OGD/R) to mimic CCH *in vitro*. We aimed to explore the effects of D3NB treatment on hippocampal neuronal death after CCH as well as its underlying molecular mechanism. We observed memory impairment and increased hippocampal neuronal apoptosis in the CCH groups, combined with inhibition of CNTF/CNTFRα/JAK2/STAT3 signaling, as compared with that of sham control rats. D3NB significantly attenuated cognitive impairment in CCH rats and decreased hippocampal neuronal apoptosis after BCCAO *in vivo* or OGD/R *in vitro*. More importantly, D3NB reversed the inhibition of CNTF/CNTFRα expression and activated the JAK2/STAT3 pathway. Additionally, JAK2/STAT3 pathway inhibitor AG490 counteracted the protective effects of D3NB *in vitro*. Our results suggest that D3NB could improve cognitive function after CCH and that this neuroprotective effect may be associated with reduced hippocampal neuronal apoptosis *via* modulation of CNTF/CNTFRα/JAK2/STAT3 signaling pathways. D3NB may be a promising therapeutic strategy for vascular cognitive impairment induced by CCH.

## Introduction

Chronic cerebral hypoperfusion (CCH) is characterized by reduced cerebral blood flow (CBF), which is induced by cerebrovascular diseases (Han et al., 2020). Studies have reported that the underlying pathophysiological mechanisms of CCH may contribute to the development of degenerative processes and cognitive decline that lead to vascular dementia (VD) and Alzheimer’s disease (AD; Duncombe et al., 2017; Li et al., 2019b). However, detailed mechanisms and effective interventions for CCH have not been fully established.

The stages of CCH are mainly characterized by pathological changes, such as oxidative stress, glial cell activation, inflammatory factor release, energy metabolism disorders, abnormal neuronal electrical activity, white matter damage (Li et al., 2019a), and hippocampal neuronal damage that result in cognitive impairment (Li et al., 2019a,b). Neuronal death is a hallmark of cognitive disorders that are induced by CCH, especially at later stages (Chen et al., 2017; Li et al., 2019a,b). These changes can also be accompanied by or lead to abnormal brain structure and function at later stages of CCH. A previous study indicated that patients with AD and VD showed typical pyramidal neuron loss in the CA1 sector of the hippocampus and that chronic “noninfarctional” hypoperfusion may latently accelerate hippocampal neurodegeneration (Nishio et al., 2010). Since the hippocampus plays a critical role in cognitive processes and the CA1 region in the hippocampus is highly vulnerable to ischemic injury (Wang et al., 2004), the molecular mechanisms underlying CCH in neuronal death in the hippocampus should be further investigated.

Recent studies have indicated that changes in neurotrophic factors are related to the pathogenesis of CCH (Du et al., 2017; Li et al., 2019b). Neurotrophic factors, which regulate neuronal survival, are promising therapeutic candidates for neurodegenerative diseases. Ciliary neurotrophic factor (CNTF) is a pluripotent neurotrophic factor, and given the importance of CNTF function in the central nervous system, it is one of the most extensively studied neurotrophic factors (Pasquin et al., 2015). CNTF is highlighted as a neurorestorative target in animal models of neurodegeneration (Davis and Pennypacker, 2018) and is a member of the interleukin-6 (IL-6) family. IL-6 and its receptors are expressed in neurons and astrocytes in various brain areas, including the hippocampus (Fang et al., 2013). The tertiary structure of CNTF allows three binding sites for its interaction with a heterotrimeric receptor composed of the ciliary neurotrophic factor receptor alpha (CNTFRa), glycoprotein-130 (gp130), and the leukemia inhibitor factor receptor (LIFRβ). CNTF binds to its receptor by first recruiting the GPI-anchored CNTFRα chain in a 1:1 stoichiometry. CNTF exerts pleiotropic activities through multiple signaling pathways including Janus kinases (JAK) and activators of transcription (STATs), leading to diverse gene transcription regulation (Pasquin et al., 2015). However, the expression and function of CNTF/CNTFRα in hippocampal neurons in CCH rats have not been extensively studied. We hypothesized that decreased CNTF/CNTFRα signaling in the hippocampus is correlated with increased neuronal death.

It is also essential to develop a safe and effective brain delivery system for neurotrophic factors. Although gene and cell therapies have been moderately successful, they are complex and expensive; therefore, the identification of drugs that target neurotrophic factors and can pass the blood-brain barrier (BBB) may be a promising therapeutic option (Li et al., 2019b).

Dl-3-n-butylphthalide (D3NB) is a synthetic chiral compound originally isolated from the seeds of *Apium graveolens* and can cross the BBB (Li et al., 2019b,c). D3NB was approved by the Food and Drug Administration of China for the treatment of ischemic stroke (Wang et al., 2019). Previous studies have indicated that D3NB has diverse neuroprotective effects in CCH, such as preserving white matter integrity (Han et al., 2019), regulating endoplasmic reticulum stress and the Shh/Ptch1 signaling-pathway (Niu et al., 2019), and promoting neovascularization (Xiong et al., 2017; Li et al., 2019c). However, few studies have focused on the role of D3NB in neuronal death (Xiong et al., 2017; Li et al., 2019b). Results from our previous study revealed that D3NB was protective against hippocampal neuron apoptosis in CCH rats and reduced cognitive impairment. Additionally, the protective effects of D3NB were associated with the regulation of the GDNF/GFRα1/Ret signaling pathways (Li et al., 2019b). Therefore, we hypothesized that the neuroprotective effects of D3NB during CCH involved the regulation of neurotrophic factors. Additionally, the role of CNTF/CNTFRα signaling in neuronal survival following D3NB treatment remains unclear.

In this study, we explored the relationship between CNTF/CNTFRα/JAK2/STAT3 signaling pathways, which are the regulatory targets of D3NB, and hippocampal neuronal death during the CCH process.

## Materials and Methods

### Animals

Specific pathogen-free, Sprague–Dawley rats (male, 250–300 g, 8 weeks old) were obtained from the Experimental Animal Center of Southern Medical University and initially housed in groups in a temperature-controlled environment (24 ± 2°C) under a 12/12 h light/dark cycle with free access to food and water. Before the experiment, the animals were raised for 4 weeks in the laboratory animal management center of Jinan University. Experimental protocols were approved by the Laboratory Animal Ethics Committee of Jinan University (No. 20160607204509). All procedures were approved by the Ethics Committee and performed following ethical standards.

### Groups and Drug Treatment

D3NB (formula: C_12_H_14_O_2_) was provided by the Shijiazhuang Pharmaceutical Group, D3NB Pharmaceutical Company, Limited (Shijiazhuang, Heibei, China).

**Figure 1:**
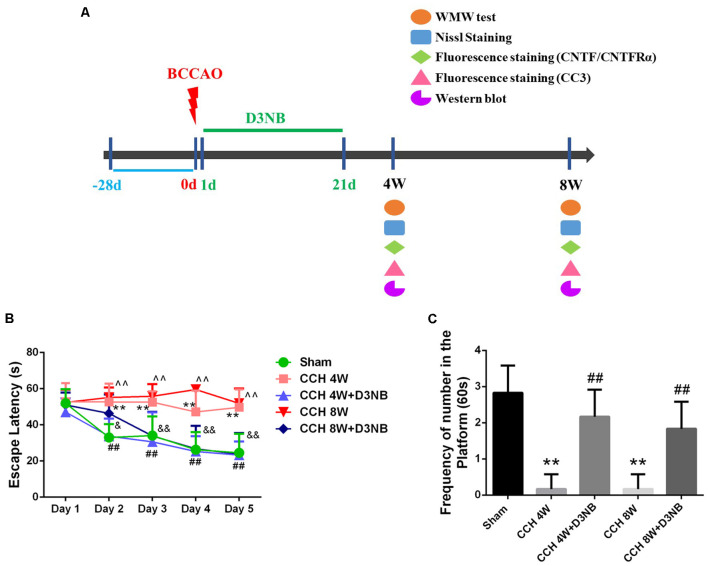
Dl-3-n-butylphthalide (D3NB) improved chronic cerebral hypoperfusion (CCH)-induced learning and memory impairments. **(A)** Animal experimental flowchart: D3NB were administrated intraperitoneally once daily from day 1–21 after CCH. Morris water maze (MWM) tests were performed at 4 and 8 weeks after Bilateral Common Carotid Artery Occlusion (BCCAO; CCH or CCH + D3NB groups). Nissl staining, fluorescence staining and western blot were detected at the indicated time point. **(B)** The escape latency (EL) of rats in the training trials of the hidden platform task (**P < 0.01 CCH 4W vs. Sham; ^##^*P* < 0.01 CCH 4W + D3NB vs. CCH 4W; ^∧∧^*P* < 0.01 CCH 8W vs. Sham; ^&^*P* < 0.05; ^&&^*P* < 0.01 CCH 8W + D3NB vs. CCH 8W; *n* = 8/group). **(C)** Frequency of platform crossing in the probe trial (**P < 0.01 CCH groups vs. Sham; ^##^*P* < 0.01 CCH + D3NB groups vs. CCH groups; *n* = 8/group). CC3, cleaved caspase 3.

For *in vivo* experiments, D3NB was dissolved in 0.9% saline with hydroxypropyl-β-cyclodextrin (1:3). As described in our previous study, D3NB-treated rats received daily tail vein injections of the D3NB solution (5 mg/kg; Li et al., 2019b). A total of 60 rats were divided into five groups (Supplementary Table 1): (1) sham group; (2) CCH 4 week (W) group; (3) CCH 8W group; (4) CCH 4W + D3NB (D3NB 4W) treatment group; and (5) CCH 8W + D3NB (D3NB 8W) treatment group. D3NB was injected intravenously through the tail vein every other day and the injections were carried out for 21 days (3 weeks). CCH groups (4W and 8W) received a solvent control (animal experimental flow chart is shown in Figure 1A).

### Bilateral Common Carotid Arterial Occlusion (BCCAO) Surgical Procedure

BCCAO operation *in vivo* is a classic and an acknowledged model for the study of molecular mechanisms of CCH and its interventions (Zhang et al., 2017; Zou et al., 2018; Li et al., 2019b; Mao et al., 2019). Rats were anesthetized with 3% sodium pentobarbital (50 mg/kg, intraperitoneal (i.p.) injection; Li et al., 2019b,c). CCH was induced using minimally invasive BCCAO operation (Farkas et al., 2007; Zhang et al., 2017; Zou et al., 2018; Mao et al., 2019). The detailed surgical procedures have been described previously (Li et al., 2019b,c; Su et al., 2019). Briefly, a ventral incision was made in the midline of the neck with a scalpel, bilateral common carotid arteries were exposed and gently separated from the vagus nerve, then permanently occluded with 4–0 silk suture in the BCCAO group. The sham rats were subjected to the same procedure without occlusion of both vessels. The body temperature of the rats was maintained using a heating lamp during the surgical procedure. After the operation, the rats were housed in an animal resource facility with *ad libitum* access to food and water.

### Morris Water Maze Test

Spatial learning and memory abilities were assessed using a Morris water maze (MWM) test (Vorhees and Williams, 2006). The MWM test was conducted in a black and circular pool that was partially filled with water at a temperature of 23 ± 1°C. The test methods have been described previously (Li et al., 2019a,b). All experimental rats performed four training sessions each day for five consecutive days. On the sixth day, the rats underwent the spatial probe test. During this test, the platform was removed, and the experimental rats were released from the quadrant opposite to the target quadrant. The rats were allowed to search for the platform for 60 s, and the frequency of platform crossings was recorded.

### Pathological Examination by Nissl Staining

After the behavioral evaluation, rats were euthanized by sodium pentobarbital overdose [100 mg/kg, intraperitoneal (i.p.) injection] and were transcardially perfused (chest ribs were dissected to expose heart tissue for perfusion) with a physiological saline solution followed by 4% paraformaldehyde. We isolated brain samples and sliced the samples into 5-μm consecutive coronal sections. Then, changes in neuronal morphology were assessed by examining the Nissl-stained histological sections. The procedure of Nissl staining was performed according to the manufacturer’s instructions and previous research (Chen et al., 2017). For each rat, three slices were randomly selected and used for immunohistochemical staining and quantitative analysis; then to get the average, three rats per group were analyzed by viewing the stains under a light microscope (Nikon 50i; Nikon, Tokyo, Japan).

### Cell Culture

Primary hippocampal neuronal cultures were harvested from the hippocampus of E16–E18 rat embryos, incubated in DMEM/F12 (Gibco, New York, NY, USA) for 4–6 h at 37°C in a 5% CO_2_ incubator. Then, the medium was changed to a neurobasal medium (Gibco, New York, NY, USA) with 2% B27 (Gibco, New York, NY, USA), and cultures were incubated for another 7 days at 37°C in a 5% CO_2_ incubator (Li et al., 2019b).

### Oxygen-Glucose Deprivation/Reperfusion (OGD/R) Model and Drug Treatment

OGD/R *in* *vitro* is used to study the mechanisms and interventions of CCH (Zhang et al., 2017; Zou et al., 2018; Li et al., 2019b; Mao et al., 2019). For the *in vitro* OGD/R experiments, the hippocampal neurons were incubated in Hank’s medium and placed in an oxygen-free culture that was induced by a hermetic bag and AnaeroPack-Anaero (Mitsubishi Gas Chemical, Tokyo, Japan) for 2 h at 37°C. Next, neurons were re-perfused and re-incubated with a neurobasal medium for an additional 48 h at 37°C in a 5% CO_2_ incubator.

The concentrations and administrative time points of the D3NB (60 μM) were based on previous studies (Li et al., 2019b). AG490 was used to selectively inhibit JAK2/STAT3 activation. JAK2 and STAT3 phosphorylation were significantly decreased by AG490 treatment at a concentration of 10 μM for 24 h in primary hippocampal neurons.

Primary neurons were randomly divided into the following six groups: (1) control group, neurons were cultured in culture medium alone without any other treatment; (2) OGD/R group, OGD for 2 h and reperfusion for another 48 h; (3) OGD/R + D3NB group, after OGD 1 h, incubation with 60 μM D3NB for another OGD 1 h plus R 24 h; (4) AG490 group, incubation with 10 μM JAK2 inhibitor (AG490) for 24 h on the 7th day; (5) OGD/R + AG490 group, incubation with 10 μM AG490 for 24 h followed by treatment with OGD/R; and (6) OGD/R + D3NB + AG490 group, incubation with 10 μM AG490 for 24 h followed by treatment with 60 μM D3NB for another 25 h under the OGD/R environment. The vehicle solution used in all the groups was a normal culture medium, which did not interfere with observation results. Except for the AG490 groups, the intervention time of the other groups was 8 days after primary hippocampal neuron culture.

### Cell Counting Kit-8 (CCK-8) Cell Viability Assay

Neuron survival was assessed using CCK-8 (Dojindo, Japan). Briefly, 10 μl of CCK-8 solution was added to 90 μl of the medium solution in each neuronal culture well of a 96-well plate and incubated for 4 h at 37°C. The absorbance was measured at 450 nm using a microplate reader (Thermo Fisher Scientific, Waltham, MA, USA).

### Immunofluorescence Staining

The hippocampus was dissected from the brain and then was serially dehydrated, embedded in paraffin, and cut into 5-μm-thick coronal sections for immunofluorescence assays. The primary hippocampal neurons that were plated on coverslips were fixed with 4% paraformaldehyde for 15 min. Next, the brain sections and neurons were washed three times with PBS and incubated overnight at 4°C in a humidified atmosphere. The following day, the brain sections and neurons were incubated with the following primary antibodies: neuron-specific nuclear protein (NeuN; 1:250; Abcam, Cambridge, MA, USA), cleaved Caspase 3 (1:250; CST, Danvers, MA, USA), CNTF (1:250; R&D systems, Minneapolis, MN, USA), and CNTFRα (1:250; R&D systems, Minneapolis, MN, USA) at 37°C for 2 h and overnight at 4°C. The following day, the sections and coverslips were washed with 0.01 M PBS for 5 min three times and incubated with the secondary antibodies (1:500, Invitrogen, Grand Island, NY, USA). Then, the brain sections were incubated with 4′,6-diamidino-2-phenylindole (DAPI; 1:1,000, Thermo Fisher Scientific, Waltham, MA, USA) at 24°C for 30 min to detect the cell nuclei. Immunofluorescence images were captured under a confocal microscope (Leica, Heidelberger, Germany), and the positive cells were counted using Image-Pro^®^ Plus (Version 6.0 for Windows™, Bethesda, MD, USA) and Image J analysis software (National Institutes of Health, Bethesda, MD, USA).

### TUNEL Staining

After receiving different interventions, the coverslips of primary hippocampal neurons were washed three times with PBS and incubated overnight at 4°C. The TdT-mediated dUTP Nick-End Labeling (TUNEL) assay (Thermo Fisher Scientific, Waltham, MA, USA) was performed according to the manufacturer’s protocol and the detailed procedures have been described previously (Li et al., 2019b).

### Protein Extraction and Western Blot Analysis

Three rats from each group were randomly selected and decapitated under anesthesia. The bilateral hippocampus tissues were quickly dissected. Total protein was extracted from the tissues or primary hippocampal neurons using RIPA lysis buffer (89901, Thermo Fisher Scientific, Waltham, MA, USA) with a protease inhibitor cocktail (1861278, Thermo Fisher Scientific, Waltham, MA, USA) and phosphatase inhibitor cocktail (B15001, Biotool, Jupiter, FL, USA). The tissues were centrifuged at 4°C for 10 min at 12,000 *g*. The protein samples were separated using 10% sodium dodecyl sulfate-polyacrylamide gel electrophoresis and electroblotted onto polyvinylidene fluoride membranes. Then, the membranes were blocked for 2 h at room temperature in a blocking buffer containing 5% fat-free milk and incubated with the following primary antibodies: anti-CNTF (1:300; R&D systems, Minneapolis, MN, USA), anti-CNTFRα (1:300; R&D systems, Minneapolis, MN, USA), anti-t-JAK2 (1:300; Abcam, Cambridge, MA, USA), anti-p-JAK2 (Tyr1007/1008; 1:300; Abcam, Cambridge, MA, USA), anti-t-STAT3 (1:300; Abcam, Cambridge, MA, USA), anti-p-STAT3 (Tyr705; 1:300; Abcam, Cambridge, MA, USA), and anti-β-actin (1:500; Abcam, Cambridge, MA, USA), which was used as a loading control (Supplementary Figure 1). The next day, the membranes were washed three times with Tris-buffered saline that contained Tween 20 and incubated with the secondary antibody (1:10,000; Abcam, Cambridge, MA, USA) for 1 h. The bands on the membranes were detected using the enhanced chemiluminescence (ECL; Thermo Fisher Scientific, Waltham, MA, USA) method and quantified using ImageJ analysis software (National Institutes of Health, Bethesda, MD, USA; Niu et al., 2019).

### Statistical Analysis

All data were analyzed using the Statistical Package for Social Science (SPSS) version 19.0 (IBM, Armonk, NY, USA). All data are presented as the mean ± standard deviation (SD). Intergroup differences in the MWM test [escape latency (EL)] were evaluated using repeated measures two-way analysis of variance (ANOVA) with Bonferroni *post hoc* test. Other data were analyzed using a one-way ANOVA when the homogeneity of variance was met, and intergroup comparisons were conducted using Fisher’s least significant difference (LSD) test. When homogeneity of variance was not met, intergroup comparisons were conducted using Tamhane’s T2 test. The prior level of significance was established at *P* < 0 05.

## Results

### D3NB Attenuates Learning and Memory Impairment Induced by CCH

According to the results from the MWM test, the spatial learning ability of rats in the different groups differed at different time points. On the first day, there was no significant difference in EL between the five groups. However, on days 2–5, the EL of rats in the CCH 4W and CCH 8W groups was significantly longer than that of rats in the sham group (*p* < 0.01, respectively). Additionally, the mean latencies of rats in the D3NB 4W and D3NB 8W groups were significantly shorter than those of rats in the CCH groups from days 2–5 (*p* < 0.05, respectively) (Figure 1B). These results suggest that the learning ability of CCH rats was impaired and that D3NB improved the learning ability of CCH rats.

During the probe trial, the frequency of platform crossings at day 6 was significantly decreased in the CCH-treated group (*p* < 0.01), while significant improvements were observed in the D3NB-treated groups (*p* < 0.01). There was no significant difference between the D3NB treated groups and the sham group (*p* > 0.05; Figure 1C). These results suggest that the spatial memory of CCH rats was impaired, and D3NB improved the spatial memory of CCH rats.

### D3NB Treatment Attenuates Pathological Death of Hippocampal Neurons in CCH Rats

The pyramidal neurons in the CA1 region of the hippocampus of rats in the sham group were tightly ranked in order. Additionally, the neurons were clear in shape and moderate in size with normal microstructure.

**Figure 2:**
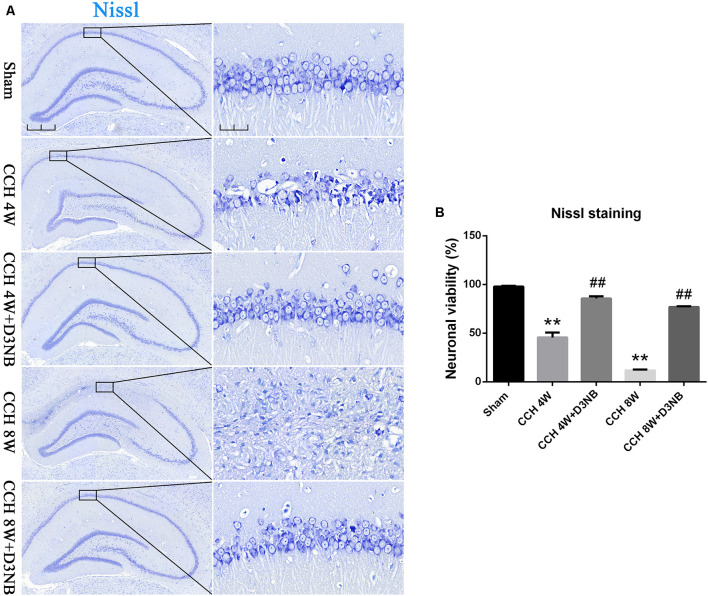
D3NB alleviated hippocampal neuronal death in CCH rats. **(A)** Sections of the hippocampal CA1 region were obtained and stained by Nissl staining in different groups (magnification ×4.0, scale bar = 500 μm; magnification ×40, scale bar = 50 μm). **(B)** Quantitative analysis of neuronal viability (**P < 0.01 CCH groups vs. Sham; ^##^*P* < 0.01 CCH + D3NB groups vs. CCH groups. *n* = 3/group, quantitative analysis of ROI, 200 μm^2^).

However, rats in the CCH 4W group exhibited distinctive pathological changes and neuronal death in the CA1 region of the hippocampus. Specifically, we observed neuronal damage and loss, and this was especially evident in the CCH 8W group. However, the administration of D3NB reversed these morphologic changes (Figure 2A). A quantitative analysis of neuronal viability is shown in Figure 2B.

### Hippocampal CNTF and CNTFRα Expression in Different Experimental Groups

**Figure 3:**
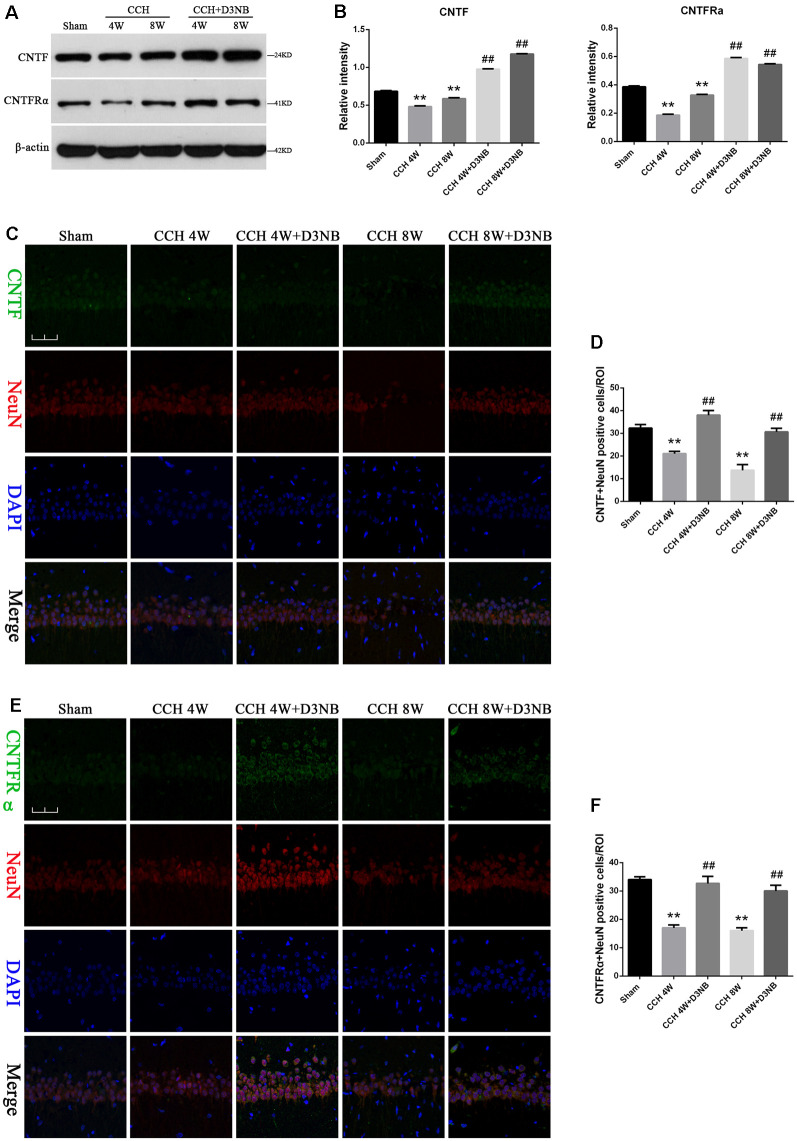
D3NB upregulated CNTF and CNTFRα expression in CCH rats. **(A)** Western blot analysis of the expression of CNTF and CNTFRα in the different groups of rats. **(B)** Quantitative analysis (**P < 0.01 CCH groups vs. Sham; ^##^*P* < 0.01 CCH + D3NB groups vs. CCH groups. *n* = 3/group). **(C,E)** Double staining of CNTF/CNTFRα and NeuN in the CA1 region of the hippocampus in different groups. **(D)** Quantitative analysis of positive CNTF-NeuN neurons *in vivo*. **(F)** Quantitative analysis of positive CNTFRα-NeuN neurons *in vivo* (magnification ×40, scale bar = 50 μm. **P < 0.01 CCH groups vs. Sham; ^##^*P* < 0.01 CCH + D3NB groups vs. CCH groups. *n* = 3/group, quantitative analysis of ROI, 100μm^2^).

Results from western blotting showed that the protein levels of CNTF and CNTFRα were significantly increased in D3NB-treated rats as compared with those in CCH rats (Figure 3A). A quantitative analysis of results is shown in Figure 3B.

To determine the localization of the CNTF/CNTFRα protein in the hippocampal neurons, we performed co-immunofluorescence staining of CNTF/CNTFRα and NeuN on brain sections from each group, and our results demonstrated that CNTF/CNTFRα expression co-localized within NeuN-positive neurons (Figures 3C,E). Positive CNTF/ CNTFRα-NeuN cells were significantly downregulated in the hippocampus of CCH rats compared with those in the hippocampus of rats that were treated with D3NB. Quantitative analysis of this data is shown in Figures 3D,F.

**Figure 4:**
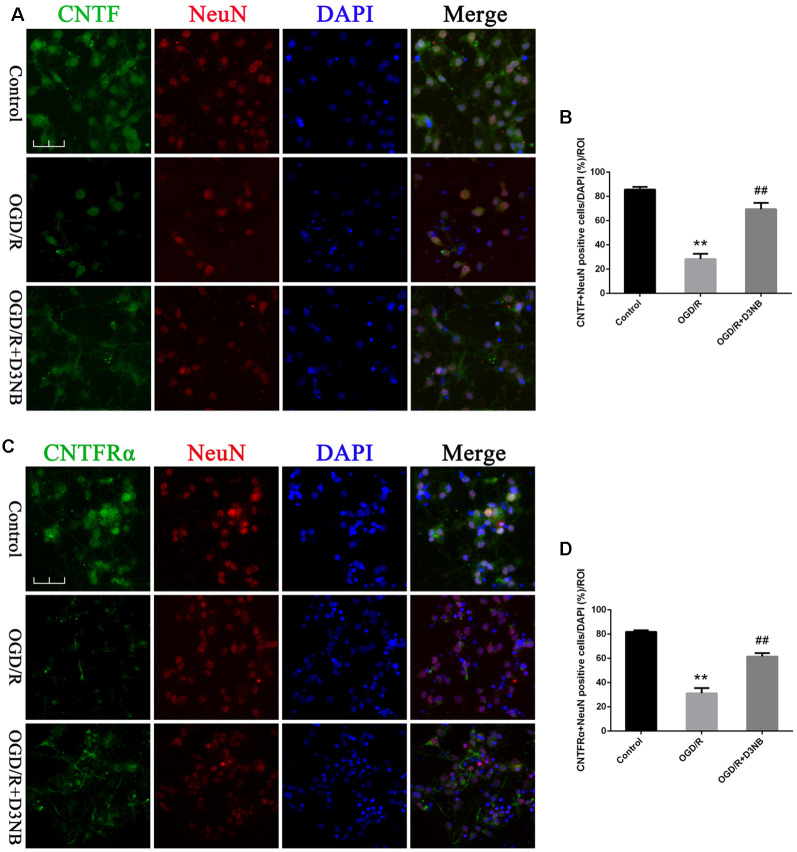
D3NB upregulated CNTF and CNTFRα expression in OGD/R-treated primary hippocampal neurons. **(A,C)** Double staining of CNTF/CNTFRα and NeuN in primary hippocampal neurons of the different treatment groups. **(B)** Quantitative analysis of positive CNTF-NeuN neurons *in vitro*. **(D)** Quantitative analysis of positive CNTFRα-NeuN neurons *in vitro* (OGD/R, OGD 2 h/R 48 h. Magnification ×40, scale bar = 50 μm. **P < 0.01 OGD/R groups vs. Control; ^##^*P* < 0.01 OGD/R + D3NB groups vs. OGD/R groups. *n* = 3/group, quantitative analysis of ROI, 200 μm^2^).

*In vitro*, although OGD/R caused a loss of positive CNTF/ CNTFRα-NeuN hippocampal neurons, D3NB (60 μM) significantly mitigated this damage caused by the OGD/R environment (Figures 4A–D).

### D3NB Treatment Activates the JAK2-STAT3 Pathway in the Hippocampus of CCH Rats

**Figure 5:**
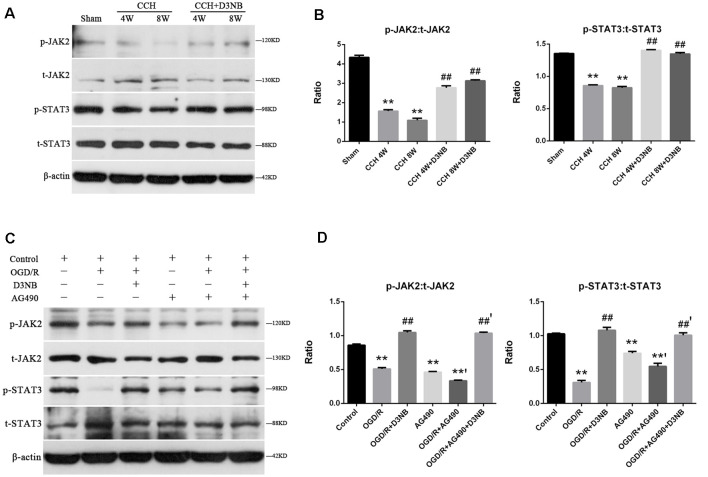
D3NB activates the JAK2-STAT3 pathway in the CCH rats and OGD/R environment *in vitro*. **(A)** Western blot analysis of the expression of t-JAK2, p-JAK2, t-STAT3, and p-STAT3 in the different groups of rats. **(B)** p-JAK2:t-JAK2 and p-STAT3:t-STAT3 ratio were decreased in the CCH-treated groups but increased in the D3NB-treated groups (**P < 0.01 CCH groups vs. Sham; ^##^*P* < 0.01 CCH + D3NB groups vs. CCH groups. *n* = 3/group). **(C)** Expression of t-JAK2, p-JAK2, t-STAT3, and p-STAT3 in primary hippocampal neurons after treatment with D3NB and/or AG490 after exposure to OGD/R *in vitro*. **(D)** Quantitative analysis of p-JAK2:t-JAK2 and p-STAT3:t-STAT3 ratio in different groups *in vitro* (OGD/R, OGD 2 h/R 48 h. **P < 0.01 compared to Control; ^**’^*P* < 0.01 OGD/R + AG490 vs. AG490; ^##^*P* < 0.01 OGD/R + D3NB vs. OGD/R; ^##’^*P* < 0.01 OGD/R + D3NB + AG490 vs. OGD/R + AG490. *n* = 3/group).

Western blotting was also performed to detect the expression of t-JAK2, p-JAK2, t-STAT3, and p-STAT3. As shown in Figure 5A, during CCH development, the ratios of p-JAK2:t-JAK2 and p-STAT3:t-STAT3 were significantly upregulated in all of the treatment groups as compared with those of the CCH rats (*p* < 0.01; Figure 5B).

To further confirm the role of JAK2 and STAT3 in D3NB-induced neuroprotection, neurons were treated with AG490, which is a JAK2 signaling inhibitor. Following treatment with AG490, the expression levels of phosphorylated JAK2 and STAT3 in neurons were suppressed as compared with those in the OGD/R plus D3NB group (Figures 5C,D).

### D3NB Decreases Apoptosis of Hippocampal Neurons Induced by CCH and OGD/R

**Figure 6:**
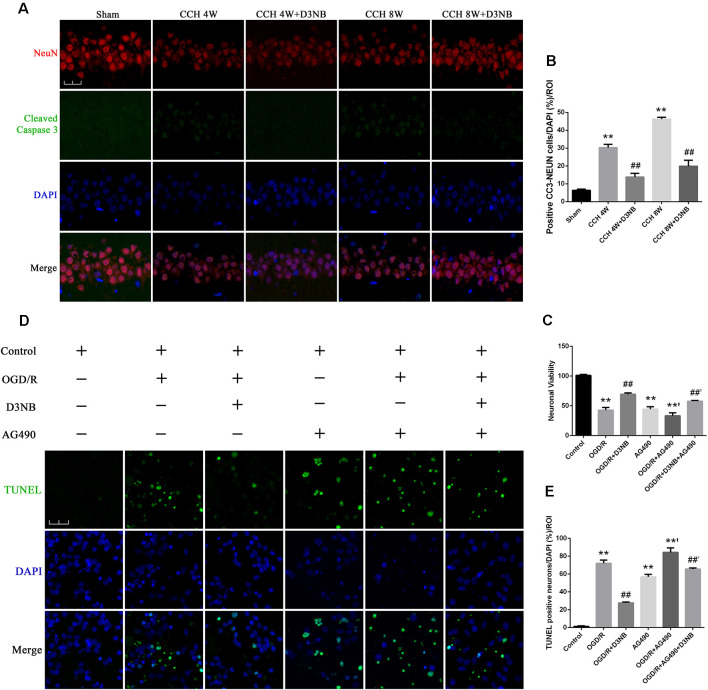
D3NB protects hippocampal neurons by antagonizing the action of apoptosis in the CCH rats and OGD/R environment *in vitro*. **(A)** Double staining of NeuN and cleaved Caspase 3 in apoptotic neurons in the CA1 region of the hippocampus in different groups. **(B)** The positive cell numbers were quantified by ImageJ Software (magnification ×60, scale bar = 30 μm. **P < 0.01 CCH groups vs. Sham; ^##^*P* < 0.01 CCH + D3NB groups vs. CCH groups; *n* = 3/group, quantitative analysis of ROI, 200 μm^2^). **(C)** Cell viability was measured using the CCK-8 method following treatment with AG490 and/or D3NB after exposure to OGD/R *in vitro* (OGD/R, OGD 2 h/R 48 h. **P < 0.01 compared to Control; **’*P* < 0.01 OGD/R + AG490 vs. AG490; ^##^*P* < 0.01 OGD/R+D3NB vs. OGD/R; ^##’^*P* < 0.01 OGD/R + D3NB + AG490 vs. OGD/R+AG490. *n* = 5/group). **(D)** TUNEL and DAPI staining of primary hippocampal neurons in the control, OGD/R, OGD/R+D3NB, control+AG490, OGD/R + AG490, and OGD/R + AG490 + D3NB groups. **(E)** Quantitative analysis (OGD/R, OGD 2 h/R 48 h. Magnification ×60, scale bar = 30 μm. **P < 0.01 compared to Control; **’*P* < 0.01 OGD/R + AG490 vs. AG490; ^##^*P* < 0.01 OGD/R + D3NB vs. OGD/R; ^##’^*P* < 0.01 OGD/R + D3NB + AG490 vs. OGD/R + AG490. *n* = 3/group, quantitative analysis of ROI, 200 μm^2^).

The apoptotic rate of neuronal cells was determined by cleaved Caspase 3 and NeuN double staining in the different groups of rats (Figure 6A). The CCH-induced neuronal cell apoptotic rate was significantly decreased in D3NB treated rats as compared with that of CCH rats (*p* < 0.01; Figure 6B).

The survival rate of primary hippocampal neurons in the OGD/R + D3NB group increased compared to that of the OGD/R group (*p* < 0.01). Although D3NB increased the survival rate of neurons to 69.4% ± 2.30, AG490 decreased the D3NB-mediated increase in survival to 57.8% ± 1.30 (*p* < 0.01; Figure 6C).

The apoptotic rate of neuronal cells was determined by TUNEL staining after the termination of the culture procedure at the designated incubation time point. The neuronal apoptotic rate of cells that were exposed to the OGD/R group was significantly increased as compared with that of cells in the control group (*p* < 0.01). Also, the neuronal apoptotic rate of cells in the OGD/R+D3NB group was significantly decreased as compared with that of cells in the OGD/R group (*p* < 0.01). However, pre-incubation with AG490 blocked the inhibitory effects of D3NB on apoptosis (*p* < 0.01; Figures 6D,E).

## Discussion

A substantial amount of evidence indicates that CCH, a chronic, moderate, and persistent reduction in CBF, leads to the development and progression of cognitive impairments (Cechetti et al., 2012; Li et al., 2019a,b); this type of dementia is classified as VD, which represents the second most common form of dementia after AD (Han et al., 2020; Leardini-Tristão et al., 2020). Despite the importance of treating CCH in aging societies, apart from improving cerebral perfusion (Li et al., 2019c) and controlling other risk factors, there is no definite treatment for CCH-related cognitive damage (Kim et al., 2020). The neuropathologic mechanisms involved in the CCH process are diverse, including neuroinflammation, oxidative stress, and white matter damage (Du et al., 2017). Irreversible hippocampal neuronal death is a hallmark of CCH (Cechetti et al., 2012; Chen et al., 2017; Li et al., 2019a) because the hippocampus plays a critical role in the cognitive processes of learning and memory consolidation (Li et al., 2019b). The hippocampal formation is highly vulnerable to ischemic injury, and significant cell loss is mainly observed in the CA1 region following cerebral ischemia (Kalaria, 2016). Clinically, patients with subcortical ischemic VD show a pattern of pyramidal neuronal loss in the hippocampal CA1 sector (Nishio et al., 2010). Neurons cannot regenerate after death, which explains how CCH contributes to the progression of cognitive impairment and why it is difficult to reverse. Thus, identifying curative interventions for neuronal death is a critical issue for researchers working on improving the CCH related cognitive impairment (Li et al., 2019a,b).

This study aimed to elucidate the molecular basis of hippocampal neuronal death in CCH and determine whether D3NB treatment could be a potentially effective intervention. We observed that the CCH process-induced cognitive impairment decreased CNTF/CNTFRα/JAK2/STAT3 signaling, and induced irreversible neuronal death in the hippocampus. This is the first study to evaluate the role of CNTF signaling in a model of CCH, and it was found that the changes in expression of this signaling pathway were related to hippocampal neuronal apoptosis. This study also provides evidence that the neuroprotective effects of D3NB are associated with the CNTF/CNTFRα/JAK2/STAT3 signaling pathways. In the current study, the administration of D3NB to CCH 4W and 8W groups’ rats improved the impaired learning and memory abilities that were observed in the MWM test. Also, when compared with the CCH rats, D3NB reversed the damage/apoptosis of pyramidal neurons in the hippocampal CA1 region. Further, the expressions of CNTF, CNTFRα, p-JAK2, and p-STAT3 were significantly upregulated after the administration of D3NB.

Given the importance of CNTF function in the central nervous system, it is highlighted as a neurorestorative target for multiple sclerosis, AD, Huntington’s disease, and amyotrophic lateral sclerosis (Pasquin et al., 2015). Neurogenesis during AD and ischemic stroke are also related to CNTF (Pasquin et al., 2015). CNTF supports the maintenance of dopamine neurons in the substantia nigra and motor neurons in the spinal cord (Kim et al., 2019). The application of exogenous CNTF to nerve lesions has also been reported to promote axonal regrowth and maturation during peripheral nerve regeneration (Fan et al., 2017). Additionally, reports of substantial weight loss following CNTF systemic administration suggests that it plays a role in metabolism and energy balance. Furthermore, CNTF exerts myotrophic activities and is also classified as a myokine that is capable of modulating osteoblast function (Pasquin et al., 2015). Results from a previous study revealed that CNTF protects against glutamate-induced neurotoxicity in dorsal root ganglion neurons *via* the regulation of the JAK2/STAT3 and PI3K/AKT pathways (Wen et al., 2017). However, the expression and potential effects of CNTF signaling on hippocampal neurons have not been studied in CCH.

D3NB is approved in China for the treatment of ischemic stroke (Wang et al., 2019). Previous studies have shown that D3NB promotes recovery after acute ischemic stroke *via* multiple mechanisms (Qin et al., 2018). Other studies report that D3NB can ameliorate memory deficits in a CCH model by enhancing hemodynamics and neovascularization (Xiong et al., 2017; Li et al., 2019c), preventing BBB leakage (Han et al., 2019), regulating the Shh/Ptch1 signaling pathway (Niu et al., 2019), or activating the antioxidative stress AKT/Nrf2 signaling pathway (Qi et al., 2018). We focused on the role of hippocampal neuronal death in CCH-induced cognitive impairment and possible interventions and found that the small molecular compound D3NB, which can cross the BBB, may target and regulate neurotrophic factors (such as the GDNF/GFRα1/Ret/AKT/ERK signaling factors) and reduce hippocampal neuronal apoptosis in CCH rats (Xiong et al., 2017; Li et al., 2019b).

Herein, the inhibitory actions of CCH on hippocampal neuronal death/apoptosis were effectively reversed by D3NB in BCCAO rats. The neuroprotective effects of D3NB may be related to the regulation of CNTF signaling, which will ultimately improve cognitive impairment. In the current study, we found that the expression of CNTF/CNTFRα protein co-localized with hippocampal neurons. Additionally, consistent with CNTF/CNTFRα protein expression, the number of positive CNTF/CNTFRα-neurons was decreased in the CCH rats as compared with that of sham rats and increased in the D3NB treated rats as compared with that of the CCH rats. Further, the downregulation of CNTF/CNTFRα strongly correlated with hippocampal neuronal death after BCCAO. We also found that the actions of CCH on hippocampal neuronal apoptosis, according to cleaved Caspase 3 labeling in the CA1 region, were partially reversed by D3NB. The JAK2/STAT3 pathway is involved in neurogenesis, proliferation, differentiation, and neuroprotection under various conditions, and CNTF signaling induces neuroprotective effect *via* the JAK2/STAT3 pathway (Pasquin et al., 2015). Our study demonstrated that the phosphorylation of JAK2 and STAT3 was significantly enhanced after D3NB treatment in CCH rats, and the JAK2 signaling inhibitor, AG490, completely neutralized the beneficial effects of D3NB on the neuronal survival rate in the OGD/R environment. Also, AG490 increased the number of positive TUNEL cells in primary hippocampal neurons after OGD/R, while the apoptosis rate of hippocampal neurons was reduced by D3NB.

The current study had some limitations. First, we did not inject CNTF directly into the hippocampus because intracerebroventricular injections could have led to cerebral injury and may not be suitable for clinical practice. Thus, future studies using new technologies to overexpress CNTF in the hippocampus will provide a better understanding of the mechanism(s) underlying neuronal death in CCH. Second, although the selection of male rats could reduce the effect of estrogen on the results, a mixed cohort might be more clinically relevant. Third, gradient concentration drug intervention strategies need to be further refined. Finally, since the progression of CCH can be longer-lasting in older age, it will be valuable to examine CNTF signaling at a more chronic point after CCH.

In conclusion, our results demonstrated that decreased CNTF/CNTFRα signaling and inactivation of the JAK2/STAT3 pathway induced by CCH were strongly associated with hippocampal neuronal and cognitive damage. Additionally, D3NB administration markedly rescued memory deficits and hippocampal neuronal death/apoptosis *via* upregulating CNTF/CNTFRα/JAK2/STAT3 signaling pathway in CCH rats. Finally, our findings suggest that D3NB may be a beneficial treatment for CCH; however, the mechanisms of action need to be characterized further.

## Data Availability Statement

The raw data supporting the conclusions of this article will be made available by the authors, without undue reservation.

## Ethics Statement

The animal study was reviewed and approved by the Laboratory Animal Ethics Committee of Jinan University (No. 20160607204509).

## Author Contributions

WL, DW, and ZZ wrote the article and contributed to neuropathological experiments. WL, DW, ZZ, and XX performed the experiments. WL and XX contributed to the Morris water maze task. ZZ and XX contributed to sample collection. WL, DW, and XX contributed to data analyses. WL, GZ, and LH designed the study and wrote the manuscript. GZ, SZ, and RZ revised the manuscript. All authors contributed to the article and approved the submitted version.

## Conflict of Interest

The authors declare that the research was conducted in the absence of any commercial or financial relationships that could be construed as a potential conflict of interest.

## Supplementary Material

SUPPLEMENTARY FIGURE 1 Original images of Western Blot in the study.

SUPPLEMENTARY TABLE 1 Animal groups and number of rats used in the research.
