# Supplementary figures and images for: Dl-3-n-Butylphthalide Alleviates Hippocampal Neuron Damage in Chronic Cerebral Hypoperfusion via Regulation of the CNTF/CNTFRα/JAK2/STAT3 Signaling Pathways
Source: Front Aging Neurosci. 2021 Jan 13;12:587403. doi: 10.3389/fnagi.2020.587403 (PMC7838126; doi:10.3389/fnagi.2020.587403)

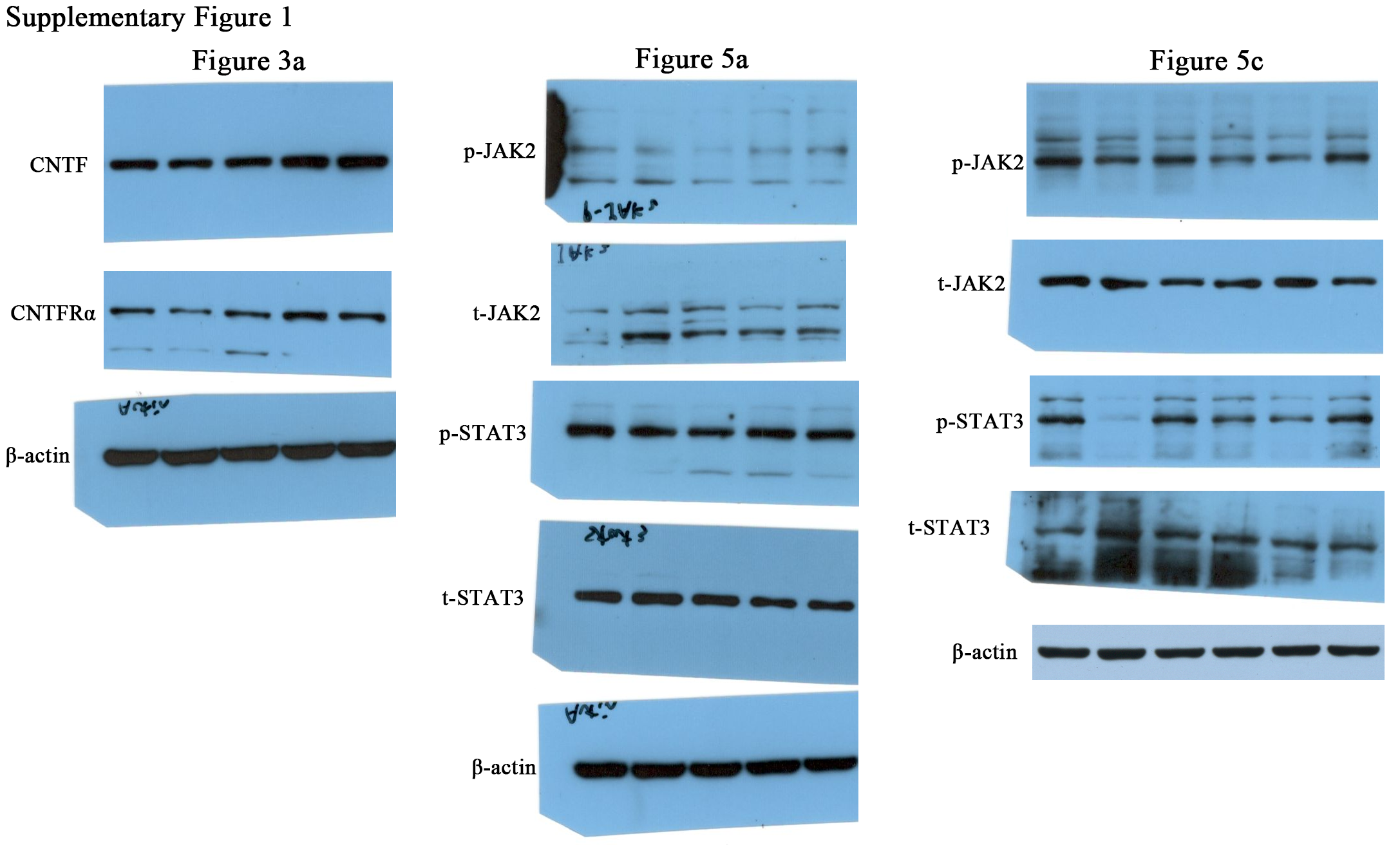

Supplement: SUPPLEMENTARY FIGURE 1 — Original images of Western Blot in the study. [file Image_1.tif]
