# Supplementary material for: Dl-3-n-Butylphthalide Alleviates Hippocampal Neuron Damage in Chronic Cerebral Hypoperfusion via Regulation of the CNTF/CNTFRα/JAK2/STAT3 Signaling Pathways
Source: Front Aging Neurosci. 2021 Jan 13;12:587403. doi: 10.3389/fnagi.2020.587403 (PMC7838126; doi:10.3389/fnagi.2020.587403)
Supplement: SUPPLEMENTARY TABLE 1 — Animal groups and number of rats used in the research. [file Table_1.docx]

| **Groups** | **Mortality** | **Exclude(dead)** |
| --- | --- | --- |
| **TOTAL**  **Sham**  **CCH**  **CCH+D3NB**  **Experiment 1: WMW test** | 0 (0/12)  11.11% (3/27)  7.70% (2/26) | 0  3  2 |
| Sham (n=8) | 0 (0/8) | 0 |
| CCH (4W, 8W) (n=8, respectively)  CCH+D3NB (4W, 8W) (n=8, respectively) | 0 (0/16)  0 (0/16) | 0  0 |
| **Experiment 2: Nissl staining** |  |  |
| Sham (n=3) | 0 (0/3) | 0 |
| CCH (4W, 8W) (n=3, respectively)  CCH+D3NB (4W, 8W) (n=3, respectively) | 0 (0/3)  0 (0/3) | 0  0 |
| **Experiment 3: Fluorescence staining (CNTF/CNTFRα)** |  |  |
| Sham (n=3) | 0 (0/3) | 0 |
| CCH (4W, 8W) (n=3, respectively)  CCH+D3NB (4W, 8W) (n=3, respectively) | 0 (0/3)  0 (0/3) | 0  0 |
| **Experiment 4: Fluorescence staining (CC3)** |  |  |
| Sham (n=3) | 0 (0/3) | 0 |
| CCH (4W, 8W) (n=3, respectively)  CCH+D3NB (4W, 8W) (n=3, respectively) | 0 (0/3)  0 (0/3) | 0  0 |
| **Experiment 5: Western blot**  Sham (n=3)  CCH (4W, 8W) (n=3, respectively)  CCH+D3NB (4W, 8W) (n=3, respectively) | 0 (0/3)  0 (0/3)  0 (0/3) | 0  0  0 |

**Table S1 Animal groups and number of rats used in the research**

A total of 65 rats were used (A total of 60 rats were available): 12 in sham group, 27 in CCH group, 26 in CCH+D3NB group. Additionally, a total of 5 rats were dead due to BCCAO surgery.
